# Perception Of Major Life Events And Depression In Mid To Later Life: Findings From A Swedish Longitudinal Study

**DOI:** 10.1093/geroni/igaf122.3055

**Published:** 2025-12-31

**Authors:** Deborah Finkel, Lawrence Sacco, Shireen Sindi, Caroline Hasselgren, Martin Hyde, Charlotta Nilsen

**Affiliations:** University of Southern California, Los Angeles, California, United States; Stockholm University, Stockholm, Stockholms Lan, Sweden; Imperial College London, London, England, United Kingdom; University of Gothenburg, Gothenburg, Vastra Gotaland, Sweden; University of Leicester, Leicester, England, United Kingdom; Jönköping University, Jönköping, Jonkopings Lan, Sweden

## Abstract

Several major life events (MLEs) that can negatively affect mental health are common in mid to later life. While MLEs are generally associated with depression, individuals’ subjective perception of such events can further explain this relationship. However, there is a lack of longitudinal studies on perception of MLEs particularly with samples including older adults. This study evaluated how MLEs and their perception are related to depression symptoms in mid to later life. The role of individual factors (e.g. sex and age) in shaping any effect of MLEs on depression was examined. Data from five waves (2010-2018) of the biennial Swedish Longitudinal Occupational Survey of Health (SLOSH) were analyzed using linear random effects regression models. The Symptom Checklist-core depression (SCL-CD6) scale was used to measure levels of depression symptoms. Respondents were asked whether in the previous two years they experienced a series of MLEs (i.e. the illness or accident of a partner; death of a partner; death of a relative or friend; marital separation; partnership formation) as well as their appraisal for each event. Analyses were adjusted for potential confounders. Results indicate that most MLEs were associated to subsequent higher levels of depression to varying extents, with bereavement being the strongest predictor and partnership formation not being associated. Perceiving any MLE as more important increased the strength of the relationship. Significant effects were found also for partnership formation once individual perception was included. Findings underscore the importance of supporting individuals in later life during transitions and key life events.

